# Systemic Overview of Microstrip Patch Antenna’s for Different Biomedical Applications

**DOI:** 10.34172/apb.2021.051

**Published:** 2020-07-01

**Authors:** Govind Arora, Paramjot Maman, Ameya Sharma, Nitin Verma, Vivek Puri

**Affiliations:** ^1^Chitkara College of Pharmacy, Chitkara University, Punjab, India.; ^2^Parexel International Limited, Mohali, Punjab, India.; ^3^Chitkara University School of Pharmacy, Chitkara University, Himachal Pradesh, India.

**Keywords:** Patch antenna, Biomaterial for Antenna, Biomedical application, Cancer, Neurodegeneration

## Abstract

Timely diagnosis is the most important parameter for the detection and hindrance with tissues (infected). Many conventional techniques are used for the determination of the chronic disease like MRI, X-ray, mammography, ultrasound and other diagnosing methods. Nevertheless, they have some limitations. We epitomize between 4 and 34 % of all carcinogenic tissues are lacking because of weak, in adequate malignant/benign cancer tissue on the contrary. So, an effective alternative method is the valid concern in the field of medical right now. Imaging with the help of patch antenna to detect chronic disease like breast cancer, oxidative stress syndrome etc. it has been proved to be a suitable potential method, and there are many works in this area. All materials have different conductivity and permittivity. With the help of these antennas, a 3D tissue structure which has different conductivity and permittivity is modelled in high-frequency structure simulator through finite element method which resolves electromagnetic field values and a microstrip patch antenna operation process. As compared with conventional antennas, micro strip patch antennas have enhanced benefits and better prospects. An integrated Antenna plays an important or crucial role for supporting many applications in biomedical, commercial and in military fields. The Antenna designed for these applications should be wideband, not sensitive to the human body. In this present review, the precise application of the Antenna in different biomedical aspects is considered. Furthermore, the author has also discussed the analytical results using simulation models and experimental results for some of the significantdisease.

## Introduction

The rapid advance in the fields of integrated circuits and micro-electromechanical systems has led to the miniaturization of various sensors and chips. Antennas have long been used in many medical applications including, microwave imaging, medical implants, hyperthermia treatments, and wireless wellness monitoring. In general, antenna is a type of receiving or transmitting device which can receive and send electromagnetic waves. Antennas are the devices which are used to convert the signals received through radio frequency into the signs of electromagnetic waves.^1^ Till date, different types of antenna are discovered which are used in variousmedical and pharmaceutical applications. These transmitting waves are electromagnetic radiation which has frequencies ranging between 300 MHz to 300 GHz. Now, these waves have unique properties which make them useful in medical purposes. Some of these fundamental properties include penetration, absorption and reflection.^2-4^ The high frequencies (short wavelengths) of these radiations are helpful in many medical aspects like hyperaemia, non-contact diagnosis, treatment in different medical diseases including various types of cancer etc. When these electromagnetic waves penetrate in the living body, then these waves encounter different environments in different body parts and hence produces varying degrees of attenuation,^5^ polarization,^6^ phase shift,^7^ and dispersion extra.^8^

Some of the types of these antennas include aperture antenna, reflector antennas and lens antennas, patch antennas, printed antennas extra. Among these printed antennas, are the ones who have fabrications created by the photolithography technique. The most basic and most advanced version of printed Antenna is a microstrip antenna. In these microstrip’ antenna’s, microstrip patch antenna (MSPA) is most widely used for medical purposes because of various advantages over another type of antennas.

Some of the advantages of MSPA includes(1)These antennas are available in different shapes like triangular, circular, rectangular, elliptical extra and hence can be used in various body parts depending upon their way (2) These antennas have a low profile, inexpensive and simple manufacturing technique as they are made by using printed circuit technology (3) Mechanically robust (4) Compatible in both planar and non-planar surfaces (5) Compatible with MMIC (Monolithic microwave integrated circuit) designs (6) Smaller in size and lighter in weight (7) Used in medical detection of affected tissues (cancerous tissues) (8) Thin profile configuration design and not sensitive to human skin (9) No cavity backing, maintenance and installation required.^9-13^ Because of these advantages, these days, we can see an increased interest in the field of electromagnetic waves used in medical therapy and diagnosis. In the treatment of some of the malignant tumours, we have seen that local and whole-body hyperthermia can successfully kill the malignant tumour tissues.^14^ Microwave energy can be used for inducing rapid hyperthermia. However, heating of deep laying tissues is still tricky by using these wavers. Moreover, the heat of a large volume of fabrics is also a challenging task by using these waves.^15^ In general, these waves have proven to be effective against deposition of energy in the affected part of skin, or the affected tissue volume (for example in the muscle, veins, organs extra) resulted in the excitation of tumour cells and hence killing of these cells due to high temperature. For ideal treatment, these waves should show minimum leakage of energy outside of the impacted area so that no killing of normal and healthy cells occurs.^16^

These waves can also be used in imaging technique, for example, Microwave breast imaging. The microwave imaging can detect early breast tumours without any use of harmful radiations. However, the antennas used are required to be immersed in liquid medium matching with biological fluids. The use of these antennas sometimes makes the system complicated, bulky, expensive and impractical. Hence, here also, to remove the complications and bulkiness caused by conventional antennas, we can use the planar and small form of microstrip antennas. These microstrip antennas have improved efficiency and practicality because of their flexible design and better ability to operate the affected tissues in the skin.^17,18^

In this review article, we will discuss the use of a MSPA in the human body and how this will work in detection and prevention event treatment of the diseases.

## MSPA (Microstrip Patch Antenna)

A MSPA consists of any planar or non-planar geometric conducting patch on one side and a ground plane on the other side. In these antennas, two metallic overlaying plates are used. One is usually more significant than the other plate. Both these plates have a dielectric sheet in between them.^14^ Most commonly, these antennas have a white and black plastic sheet. This plastic sheet not only protects the antenna but also it becomes easy for the antenna to mount on it. These antennas are usually flat, light weighted and have thin surface and hence showed better patient compliance.^19^

In these days a lot of research work is going on for the implantation of different biomedical devices like these MSPAs and others inside the human body. In order to achieve better communication through implanted antennas,^20^ there is a requirement of put antenna in both inside and outside the body. This is necessary as if one antenna sends the signal, and then the other one can receive it. This is very beneficial as there will be no need of using wires by piercing the human skin. This prevents various other infections during medical diagnosis, which can be caused by penetrating wires inside the body.^21^

The medical implant communication service (MICS) is a medical committee who decides which frequency bands and power levels are used for the antenna. Before the implantation of any antenna in body, the frequency and power acceptance is essential from the MICS.^22^ Usually, MICS operates within the band range of 402-405 as it is considered safe for the human body. The power level accepted by MICS is up to 25 Watt. These ranges are allowed only if implantation of the microstrip antenna is applicable in the body.^23^ However, if in place of implantation we are concerned with the diagnostic purpose, then MICS may allow the increase in band range, power and frequency of antenna as it is more beneficial for the human body if used for a short duration. While for long-duration above-mentioned band limits are accepted. So, the usual frequencies that can be allowed for industrial, medical and scientific applications are similar for most of the antennas (around 27 MHz, 477 MHz and 2.4 MHZ) depending on the duration of using antenna.^24^

Sometimes, the researchers have to face the frequency difference in the antennas used. For example, if the antenna has frequency 27 MHZ outside the body, then it will not give the same rate inside the body. Even, if we place such antennas inside the body, they do may have aspects of variable frequency (frequency level changes). So such antennas are usually termed as resonating antennas which do not have constant frequency level inside and outside the body.^25^ So, for such microstrip resonating antennas the band is accepted at 2.45 MHz so that minor alteration in frequency level will not impact the human body. When such antennas are placed inside the body surface, then their frequency level decreases as compared to 2.45 MHz (which it was designed for outside the body). The reduced frequency may be about 0.8 MHz. This decrease in rate maybe because of the high conductivity of the tissues as well as due to high permittivity of the muscle tissues.^26^ However, no one can judge the reduction level in frequency as it can be 0.8 MHz or can be more or less than that of 0.8 MHz; this is because the permittivity and conductivity of tissues depend on body to body and on the body part where the antenna is to be used. Researchers are working on it to fix this problem. Recently, it was noticed that this problem could be reduced by a certain extent by using the hexagonal shaped patch. The hexagonal micro-patch can help maintain the constant frequency of the antenna.^27^

The different body parts and body conditions have different physical properties (Table 1) and hence the frequency and power properties of antenna to be used changes concerning body conditions.^28-31^

**Table 1:** Different physical properties occurring in the brain (that is at different parts of the brain)^28-31^

**Physical properties**	**Center of brain**	**Grey matter**	**White matter**	Cerebrospinal liquid
Conductivity	1.15 S/m	1.8 S/m	1.2 S/m	3.4 S/m
Density	1.040 kg/m^3^	1.038 Kg/m^3^	1.038 kg/m^3^	1.007 kg/m^3^
Specific heat	3900 J/Kg.K	3680 J/kg.K	3600 J/kg.K	4000 J/kg.K
Blood flow rate	0	9×10-^6^ m^3^/kg.s	9×10-^6^ m^3^/kg.s	9×10-^6^ m^3^/kg.s
Thermal conductivity	0.5 W/m.K	0.5 W/m.K	0.5 W/m.K	0.6 W/m.K
Relative permittivity	38	50	36.2	66

Note. Here the units stand as: (S/m: Siemens per minute; W/m.K: Watt/meter Kelvin; m^3^/Kg.s: meter^3^/kilogram. Second; J/kg.K: Joule/kilogram. Kelvin; kg/m^3^: kilogram/meter^3^).

Per the above table, we can see different physical properties (conductivity, thermal conductivity, relative permittivity, density, blood flow rate and specific heat) at different parts of brain. Similarly, in other parts of the body, like lungs, kidneys, liver extra, there will be such different physical properties. The choice of microstrip antenna to be used is dependent on the conditions of that body part. And hence, the parameters of the microstrip antenna should be changed.

Other than this another issue of using the patch antenna can be its biocompatibility with the body. The antenna implanted should not impact the basic body parameters and should not harm the body tissues. Hence the antenna should be fabricated with the biocompatible materials which will not show any adverse effects inside the body. Usually, the material tungsten is used for making the ground plane of the antenna as it does not affect the body tissues.^32^ The basic properties like superior fracture and resistance to fatigue have made the tungsten metal as a metal of choice for implantable antennas. It also does not show any corrosion when inserted inside the body. Other than this, silicon can also be used as a substrate for making the antenna.^33^ Silicon is a semiconductor material which maintains its basic mechanical and electrical properties inside the body. It does not show any inflammatory and toxic responses inside the body. Silicon is used because it has a reliable high volume of fabrication, abundance in nature, ease of fabrication and exceptional physical properties. Silicon made antenna is most commonly used in breast implants and in prosthetics which can be used for better facial characteristics.^34^

The antenna performance depends on the thickness of the substrate metal used. The thin the substrate metal used, the better will be the performance of the antenna and the thick substrate metal used, the less will be its performance. The dielectric constant of the silicon metal can be reduced by removing the underneath silicon from the patch. This resulted in reduced thickness and creation of air mixed silicon. This air mixed silicon has predetermined dielectric constant value as needed.^35^ Some bio-medical applications of using MSPA are:

### Telemedicine application

Individually, in the case of tele medicines antenna are being operated at a frequency of 2.45 GHz. Mostly the preferred microstrip antennas are the wireless body area networks.^35^ So, these new and proposed antennas somewhere or the other gained popularity amongst the antenna use amongst the researchers due to the reason of higher gain and front to back ratio compared to the other antennas, in addition to the semi-directional radiation pattern which is preferred over the Omni-directional design to overcome unnecessary radiation to the user’s body and satisfies the requirement for on-body and off-body applications. So, the ideal condition considered for the appropriate functioning of the antenna is 6.7 dB and a F/B ratio of 11.7 dB and resonates at 2.45 GHz is suitable for telemedicine applications.^36^

### Medicinal applications of the patch

Microwave radiation is indeed the most effective way to trigger hyperthermia in the treatment of malignant tumours. The development of the specific radiator to be used for this function should be lightweight, easy to handle and durable. Only the patch radiator needs to satisfy these requirements.^37^ The original prototypes of the microstrip radiator for triggering hyperthermia are based on the dipoles and rings printed on the S-band. Later on, the model is based on the circular microstrip disk on the L-band. There is a simple operation that goes on with the instrument; two coupled Microstrip lines are separated by a flexible separation which is used to measure the temperature within the human body. A versatile patch applicator can be seen from the figure below, which is 430 MHz.^38^

### Sensing directly using site-directed mutagenesis

The addition of artificial molecular detection sites will massively expand the sensing capabilities of nanopores. Four histidines were integrated into the P-hemolysin sub-unit, which was then transformed into a heteromeric pore composed of six wild-type and one mutant P-hemolysin sub-units (WT64H1).^39-41^ The resulting pore was capable of detecting two or more divalent metal ions simultaneously. Three aromatic (Phe, Trp, Tyr) Met113-haemolysin mutations can be identified by TNT. In phi29 nanopore, ethane, thymine and benzene with reactive thioester moieties were clearly distinguished from K234 by binding to mutant cysteine. Fingerprints of different peptides and oligomeric peptide structures can be found by phi29 channel based on distinctive current blockage signatures.^39^ One-way peptide translocation has been identified in both SPP1 and phi 29 motor channels. Also, the position of the channels injected into the membrane was regulated by the hydrophobicity/hydrophilicity of either the terminal ends of the protein.^40^

### Sensing via probes introduced through covalent linker

One end of a long 3.4 kDa polyethylene glycol chain cross-labelled with biotin was added to the S106C-α haemolysin mutant to detect streptavidin. Thrombin can be identified by hybridization of DNA aptamer to N17C-sub-hemolysin mutant.^39^ Likewise, modified ClyA nanopore consisting of DNA aptamers with activated thiol can differentiate between human and bovine thrombin given 86% sequencing similarity. Also, binding kinetics can be observed as shown by binding of tetravalent lectin to a ligand-derived Gal-b-1,3-GalNac shaped a-hemolysin pore.^39^ Similarly, a ligand, PKIP5-24, had been attached to the trans-entry of α-hemolysin at position C129 to study the binding of cAMP-dependent protein kinase to the ligand.^41^ In some other study, a single photolabile carbamate group was attached to the inner wall at Thr117Cys of α-hemolysin.^42^ Upon UV illumination, the intermediates of decomposition can be detected because the current depends on the inner wall.^43^ Similarly, azobenzene can be attached via a di-sulphide bond to Thr117Cys α-hemolysin to study light-induced isomerization at the single-molecule level.^40,44-46^

### Probes introduced through fusion protein expression

The introduction of new functions through the moulded expression of the encoded gene sequence of proteins is challenging.^47^ One method is to combine physiologically preserved protein regions with generally independent protein domains. In one study, 5-007-hemolysin was introduced to the functional elements of co-chaperonin GroES to study interaction with GroEL at a single-molecule level.^48^ The fused GroES flexible loops showed correct folding with enzymatic activity counteracting GroEL-assisted protein folding as native GroES.^49,50^ In another study, the epithelial cell adhesion molecule (EpCAM) peptide was co-expressed with the phi29 C-terminus connector. Binding of EpCAM antibodies sequentially to each stepwise-induced peptide probe in the current.^51^ The kinetics of the probe-antibody docking can be studied in real-time at a single molecule level through characteristic current signatures. Importantly, the EpCAM antibody can be distinguished in the presence of serum or non-specific antibody.^52^

### Sensing with non-covalent adaptors

Host–guest interactions can provide an interface in the lumen of protein nanopores. Another group of specific analyte binding adapters are ring-shaped molecules, such as β-cyclodextrin, which has a hydrophobic internal cavity and a hydrophilic exterior. Possible hosting sites of β-cyclodextrins at the inner end of the β-barrel were enhanced by the mutagenesis-directed site of Met113, Lys147 and Glu111. The binding affinity was improved 104-fold relative to the wild type, resulting in a prolonged period of residence of approximately ten seconds. Organic ligands and medications are retroactively bound to the connector and culminated in analyte with signature finger blockade prints.^48^

### Sensing with covalent adaptors

β-Cyclodextrin may also be covalently attached to Met113Cys-haemolysin by a disulphide bond. This is particularly useful for single-molecule exonuclease DNA sequencing, which requires the sustained role of a molecular adapter. The orientation of the adapter can also be fixed so that the analytes can activate only one of the entrances to the cyclodextrin cavity. For example, unlabeled nucleoside 50-monophosphate and methylated cytosine can be identified by a covalently attached adapter with an accuracy of 99.8%.^48^

## Comparison of the design considerations of MSPAs for bone, muscles and skin

As we have discussed above that the implantable antenna’s design strongly vary concerning the dielectric properties of different body tissues.^53^ As per the body part conditions, various parameters and different design considerations should be taken for designing the microstrip antennas, which is most suitable and non-toxic at the affected part of the body. The antenna design considerations which should be taken into account for designing antennas to be used in skin (muscles) and bones.^54,55^

### For skin

For skin, the dielectric permittivity is found to be similar to that of muscles. Hence, lesser miniaturization of the antennas is required. The final results showed narrower bandwidth and therefore more minor mistuning difficulties in finding the actual time-variant at that part of body.^56,57^

The skin implants should also have to be smaller in its dimensions. And hence, the resonance of the inserts can usually be found less than 350 MHz. But since the skin permittivity is high so there is no resonance loss and therefore can give better results.^58^

**Figure 1:**
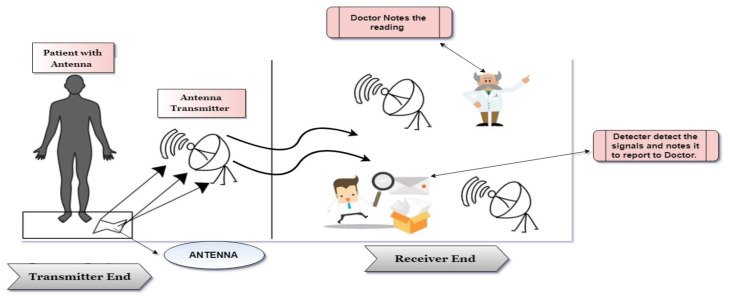
Transferring of signal from transmitter to receiver.

When the propagation signals are sent through the skin implanted inserts inside the body to the external which is placed outside the body, and then more losses can be seen in the propagation signal because of the extra layers of skin.^59^ This means that the transmitter and the receiver placed under the same sink conditions (one inside the body and other outside the body) should be placed at a shorter distance. They cannot communicate where the range is long between transmitter and receiver (the signals transferred from transmitter to receiver shown in Figure 1).^60^

The specific absorption rate for the Skin is higher as compared to bones. This is because of the high volume density of the skin. Hence, the design of the MSPAs should be adjusted as per need. Ideally, the implantable antenna should have the slightly high signal waves as required outside the body because deep skin will absorb the signals (Table 2) and hence, signal losses are possible before the message reaches to the receiver.^61,62^

**Table 2:** Different types of implantable antennas with their dimensions used in skin

**Antenna type**	**Frequency**	**Dimensions of antenna (mm^3^)**	**Model used**	**References**
PIFA	402 MHz	32 × 24 × 4	Skin	63
Dipole	402 MHz	16.5 × 15.7 × 0.27	Skin	64
PIFA	402 MHz	22.7 × 49 × 1.9	Skin	65
Dipole	542 MHz	27 × 14 × 1.27	Skin	66
PIFA	402 MHz	8 × 8 × 1.9	Skin	67
Monopole	402 MHz	18 × 16 × 1	Skin	68
Slot	402 MHz	10 × 11 × 27	Skin	69

### For monitoring different pathological changes relating bones

The bone implants have larger dimensions as compared to the implants used for skin and muscles. The larger size of bones is required to achieve the functionalities of the replaced supportive part of the bones. Because of the large size of the inserts, the resonance can be reached around 400 MHz.^70^ Since, and the bones show better resonance because of the larger size of the implantable inserts despite smaller permittivity of bones.^71^ While for muscles and skin, the insert dimensions are more minor but permittivity is high, so their resonance loss becomes lesser.^72^

For bones, dielectric permittivity is found to be smaller than that of muscles and skin. Hence, more miniaturization of the implantable antennas is required.^73^ So that the final results can show narrower bandwidth and hence lesser mistuning difficulties in finding the actual time-variant at that part of body.^74^

When the propagation signals are sent through the bone implanted inserts inside the body to the external which is placed outside the body, and then more losses can be seen in the propagation signal because of the extra layers of bone.^75^ Also, a reflection of the signals can be seen in the boundaries between the bone and muscles.^76^ This means that the transmitter and the receiver placed under the same sink conditions (one inside the body and other outside the body) should be placed at a shorter distance.^77^ They cannot communicate where the distance is long between transmitter and receiver.^78^

The specific absorption rate for the bones is smaller in amount. This is because of the high mass density of the bones.^79^ Hence, the design of the MSPAs should be adjusted as per requirement. Ideally, the implantable antenna should have the appropriate signal waves as required outside the body because bones will not absorb the signals. Hence, there will be no signal loss.^80,81^

### For monitoring different pathological changes relating muscle disorders

For muscles, the dielectric permittivity of the muscles is more colossal. Hence, lesser miniaturization of the antennas is required. The final results showed narrower bandwidth and therefore lower mistuning difficulties in finding the actual time-variant at that part of body.^82^

The muscle implants should be smaller in its dimensions, and hence, the resonance of the inserts can usually be found less than 350 MHz.^83^ But since the muscle permittivity is high so there is no resonance loss and therefore can give better results.^84^

The easiest way to reduce the dimensions, size and weight of the implantable antennas, we can use the high permittivity dielectric substances. The high permittivity of the antennas could reduce the wavelength and thus helps produce the resonant frequency as compared to that of lower frequency.^85^

When the propagation signals are sent through the muscles implanted inserts inside the body to the external which is placed outside the body (Table 3, 4, 5), and then more losses can be seen in the propagation signal because of the extra layers of muscle.^90^ Also, a reflection of the signals can be seen in the boundaries between the bone and tissues.^91^ This means that the transmitter and the receiver placed under the same sink conditions (one inside the body and other outside the body) should be placed at a shorter distance. They cannot communicate where the distance is long between transmitter and receiver.^92^

**Table 3:** Various materials with different dielectric constant values

**Material**	**Value of dielectric constant**	**References**
Macro	6.1	86
ARLON 1000	10.2	87
Rogers 3210	10.2	88
Rogers 3010	10.2	89
Rogers 6002	10.2	88

**Table 4:** Comparison of permittivity and conductivity in bone, muscles and skin

**Tissues**	**Permittivity**	**Conductivity**
Muscle	52.7	1.7
Skin	38	1.4
Bone	18.5	0.8

**Table 5:** Different types of implantable antennas with their dimensions used in Muscles

**Antenna type**	**Frequency**	**Dimensions of antenna (mm^3^)**	**Model used**	**References**
PIFA	402 MHz	26.6 × 19.6 × 6	Muscle	21
PIFA	402 MHz	28 × 24 × 6	Muscle	94
Cavity slot	2.4 GHz	1.6 × 2.8 × 4	Muscle	95
PIFA	402 MHz	22.5 × 22.5 × 2.5	Muscle	96

The specific absorption rate for the muscles is higher as compared to bones. This is because of the high volume density of the muscles. Hence, the design of the MSPAs should be adjusted as per need. Ideally, the implantable antenna should have slightly high signal waves as required outside the body because muscles will absorb the signals and hence, signal losses are possible.^93^

## Some significant disease being focusedon treating by the patch antennas

### Medical diagnosis using microwaves

The primary applications for medical diagnosis are in the detection of stroke, water accumulation in the human body and the most important one is breast cancer which is the most prevalent form of cancer among women [FABC07].^97,98^ Approximately one million ‘women’s around the world are suffering from the breast cancer [MJ13].^99,100^ Therefore, technologies with high sensitivity and accuracy to detect the presence of tumours are required. An almost pain-free assessment with a portable apparatus and a short examination time is especially desirable for the detection of early-stage breast cancer.^101^

### For monitoring different pathological changes relating breast cancer

A patch antenna is accessible, which has already been designed to radiate into the human breast tissue. The antenna has been potentially explained by practical calculation and simulation to have a wide input bandwidth, an excellent front-to-back ratio and stable radiation patterns. A low-profile, wide-band patch antenna model for breast tumour detection has been addressed. This antenna was designed to emanate directly into a dielectric medium that has the same dielectric properties as breast tissues.^101^ Have proved that this model of the mounted patch antenna provides a bandwidth of approximately 77 per cent and a beam diameter of roughly ± 400 in the 0= 00 plane and ± 300 in the 0 = 900 planes at 6.5 GHz, measured using FDTD in the 0 = 9.8 range. Measured radiation properties in the synthesized dielectric medium have also been obtainable and confirm the calculated pattern characteristics of the antenna.^102^ The time-domain characterization of the antenna indicates that this antenna is suitable for the short pulse radar application. A full slot double-sided microstrip antenna with fork feed developed for radar-based microwave imaging has beenpresented.^103^ The proposed antenna has an ultra-wide bandwidth of 3 GHz, which is more than 0.2 times the central frequency and a VSWR of 1.3539, which is less than two times the maximum bandwidth. A directive antenna with directivity of 4.0180 dB is used for radar-based imagery. These are the details obtained from the microstrip antenna for breast tumour detection.^104,105^

### For monitoring different pathological changes relating to cerebrospinal fluid

The adjustments in dielectric properties of cerebrospinal liquid (CSF) can be used in the conclusion of cognitive diseases.^106^ The point of this paper is to research the employability of an implantable receiving wire to simultaneously work as a radiator and sensor of the dielectric properties of CSF. The radio wires endeavour limited cuts and holes as capacitors for detecting the permittivity of CSF.^107^ Three receiving wires are structured dependent on the capacitively stacked circle,^108^ corresponding split ring circle,^109^ and interdigital capacitor circle.^110^ To research if the dielectric properties of CSF are an element of age, in vitro estimations of various CSF from pigs with different ages are estimated in the sub-1-GHz band (0.1–1 GHz). The outcomes uncover a strong pairwise relationship surpassing 0.77 for permittivity and 0.83 for conductivity among tests. Consequently, the dielectric properties of the CSF do not reflect age-reliance and detecting affectability is positively not influenced by age. At long last, the implantable radio wires are created and tried in a sensible domain viz inside a piglet’s head and CSF simulants. The move in the reverberation recurrence when the permittivity is expanded by 14% at 400 MHz runs between 31–40 MHz with a relative reverberation affectability somewhere in the range of 6% and 8% for the proposed implantable reception apparatuses.^111^

## Future Prospective

These days the interest of using biomedical telemetry has significantly increased because of its beneficial applications as compared to others. The useful applications include endoscopy, various health parameters monitoring like (glucose, temperature, pressure, heart rate extra), cardiac care and many more. The use of biomedical telemetries like implantable antennas and devices increases these biomedical applications. When we compare implantable devices with wearable devices, implantable devices provide better vital signals from the body where these devices are implanted. However, the design of these implantable devices is very challenging because it consists of a collaboration of various small components. Since the implantable devices should be as low as possible in their size because they are supposed to be inserted inside the body. But when we reduce the size of the antennas, there is loss and degradation of the electromagnetic waves occurs, which ultimately affect the performance of the antenna. Hence, new techniques and structures should be investigated to tackle these issues. Ideally, the antennas should have characteristics like lightweight, relatively high radiation efficiency, wide bandwidth, small size so that can be implanted inside the body, should work in multiple bandwidths. Power saving and power transfer in wireless mode are possible if the antenna can work in various bandwidths. Although most of the characteristics mentioned here are contradictory to each other, these can be satisfied because of the A’ ‘antenna’s flexibility which enables the small-sized antenna to work same as that of large-sized antenna with respect to its radiations. The larger physical size helps in maintaining wide bandwidth. So for small A’ ‘antenna’s to have wide bandwidth, dielectric and light-conducting materials are used. This helps in reduction of the antennas weight and size.

## Conclusion

In this review article, we can see various issues related to the implantable antennas. At the same time, we have discussed some future perspective, which can help in solving these issues. Since most of the antennas are implanted inside the body so these should be designed by using biodegradable materials, this could be the main attraction point, to both patients and surgeons, because it is harmless to the body and will degrade with time without any toxic effects, hence no need of any removal surgery. Although by using the biodegradable material like silk, some implantable antennas have recently been proposed, more designs are required. Organic based materials can also be used in designing implantable antennas. Another issue with implantable antenna design is its linear polarization. This linear polarization occurs because of the single fixed position of most of the implantable antennas. However, circular polarization implantable antennas can be created which can receive power supply by rotating inside the human body. When we insert or implant the antenna inside the body, there is always a power or signal loss from the transmitter to the receiver because of the multiple layers of tissues present between the transmitter and receiver. So, to enhance the communication between the transmitter and receiver, the distance between both should be as less as possible. Thus, a future work related to interface between the antenna and mobile phone can be done and can be estimated because mobile phones are carried by every person and can be checked for the signals received through antenna. Other than these, if the work can be done for wireless power transfer, then it will also become the hot research topic as this will be very beneficial as compared to the passive implants. The cost of replacing the implants or charging the implant battery and the pain in the body, surgery charges extra can be reduced if the wireless power transfer work becomes successful in future.

## Ethical Issues

Not applicable.

## Conflict of Interest

The authors declared no conflict of interest in the manuscript.

## Acknowledgments

The authors gratefully acknowledge Dr. Madhu Chitkara, Vice Chancellor, Chitkara University, Rajpura, Punjab, India, and Dr. Sandeep Arora, Director, Chitkara College of Pharmacy, Chitkara University, Rajpura, Punjab, India for support and institutional facilities.

## References

[1] Lerosey G, de Rosny J, Tourin A, Derode A, Montaldo G, Fink M (2004). Time reversal of electromagnetic waves. Phys Rev Lett.

[2] Biswas S, Arief I, Panja SS, Bose S (2017). Absorption-dominated electromagnetic wave suppressor derived from ferrite-doped cross-linked graphene framework and conducting carbon. ACS Appl Mater Interfaces.

[3] Van Etten P, Brown RD. System and Method for Earth Probing with Deep Subsurface Penetration Using Low Frequency Electromagnetic Signals. United States Patent; 1994. US 5,357,253. inventors; Earth Sounding International, assignee.

[4] Fuchs MH, Gerszberg I. Method and Apparatus for Sensing a Condition in a Transmission Medium of Electromagnetic Waves. United States Patent; 2017. US 9,768,833. 2017. inventors; AT&T Intellectual Property I LP, assignee.

[5] Barnickel DJ, Barzegar F, Bennett R, Gerszberg I, Henry PS, Willis TM. Method and Apparatus for Reducing Attenuation of Electromagnetic Waves Guided by a Transmission Medium. United States Patent; 2017. US 9,749,013. inventors; AT&T Intellectual Property I LP, assignee.

[6] Wolf E (2003). Unified theory of coherence and polarization of random electromagnetic beams. Phys Lett A.

[7] West JB. Low-Cost One-Dimensional Electromagnetic Band Gap Waveguide Phase Shifter Based ESA Horn Antenna. United States Patent; 2007. US 7,307,596. inventor; Rockwell Collins Inc, assignee.

[8] Uhm HS, Kim HS, Park GS (2002). Dispersion relation of the electromagnetic waves propagating through a helix inserted into a magnetron-type conducting cylinder. IEEE Trans Plasma Sci.

[9] Sangam RS, Poolakkal S, Palthiya R, Nallam N, Kshetrimayum RS (2019). Dual-port, aperture coupled and tapered fed patch antenna for full-duplex ISM applications. Microw Opt Technol Lett.

[10] Sabban A (2018). New wideband compact wearable slot antennas for medical and sport sensors. J Sens Technol.

[11] Jeong MJ, Hussain N, Park JW, Park SG, Rhee SY, Kim N (2019). Millimeter-wave microstrip patch antenna using vertically coupled split ring metaplate for gain enhancement. Microw Opt Technol Lett.

[12] Zhang Y, Chen Y, Li Y, Qu K, Ren T (2020). Modeling technology of InP heterojunction bipolar transistor for THz integrated circuit. Electronic Networks, Devices and Fields.

[13] Nalam M, Rani N, Mohan A (2014). Biomedical application of microstrip patch antenna. International Journal of Innovative Science and Modern Engineering (IJISME).

[14] Strohbehn JW, Bowers ED, Walsh JE, Douple EB (1979). An invasive microwave antenna for locally-induced hyperthermia for cancer therapy. J Microw Power.

[15] Amad AAS, Loula AFD, Novotny AA (2017). A new method for topology design of electromagnetic antennas in hyperthermia therapy. Appl Math Model.

[16] Kwon S, Lee S (2016). Recent advances in microwave imaging for breast cancer detection. Int J Biomed Imaging.

[17] O’Loughlin D, O’Halloran M, Moloney BM, Glavin M, Jones E, Elahi MA (2018). Microwave breast imaging: clinical advances and remaining challenges. IEEE Trans Biomed Eng.

[18] Ma XF, Yu JG, Wan JJ (2006). Urea and ethanolamine as a mixed plasticizer for thermoplastic starch. Carbohydr Polym.

[19] Sani A, Rajab M, Foster R, Hao Y (2010). Antennas and propagation of implanted RFIDs for pervasive healthcare applications. Proc IEEE.

[20] Kaatz M, Elsner P, Bauer A (2008). Body-modifying concepts and dermatologic problems: tattooing and piercing. Clin Dermatol.

[21] Soontornpipit P, Furse CM, Chung YC (2004). Design of implantable microstrip antenna for communication with medical implants. IEEE Trans Microw Theory Tech.

[22] Islam MN, Yuce MR (2016). Review of Medical Implant Communication System (MICS) band and network. ICT Express.

[23] Yang ZJ, Zhu L, Xiao S (2018). An implantable wideband circularly polarized microstrip patch antenna via two pairs of degenerate modes. IEEE Access.

[24] Kouloulias V, Karanasiou I, Giamalaki M, Matsopoulos G, Kouvaris J, Kelekis N (2015). Theoretical analysis, design and development of a 27-MHz folded loop antenna as a potential applicator in hyperthermia treatment. Int J Hyperthermia.

[25] Thielens A, Benarrouch R, Wielandt S, Anderson MG, Moin A, Cathelin A (2018). A comparative study of on-body radio-frequency links in the 420 MHz⁻24 GHz range. Sensors (Basel).

[26] Basu S, Srivastava A, Goswami A (2013). Dual frequency hexagonal microstrip patch antenna. Int J Sci Res Publ.

[27] Pajouhesh H, Lenz GR (2005). Medicinal chemical properties of successful central nervous system drugs. NeuroRx.

[28] Inum R, Rana MM, Shushama KN, Quader MA (2018). EBG based microstrip patch antenna for brain tumor detection via scattering parameters in microwave imaging system. Int J Biomed Imaging.

[29] Mahalakshmi N, Jeyakumar V (2012). Design and development of single layer microstrip patch antenna for breast cancer detection. Bonfring International Journal of Research in Communication Engineering.

[30] Zhao Y, Rennaker RL, Hutchens C, Ibrahim TS (2014). Implanted miniaturized antenna for brain computer interface applications: analysis and design. PLoS One.

[31] Hashemi S, Rashed-Mohassel J (2018). Design and miniaturization of dual band implantable antennas. Biocybern Biomed Eng.

[32] Zhang K, Liu C, Liu X, Guo H, Yang X (2017). Miniaturized circularly polarized implantable antenna for ISM-band biomedical devices. Int J Antennas Propag.

[33] Terry RS, Tarver WB, Zabara J (1991). The implantable neurocybernetic prosthesis system. Pacing Clin Electrophysiol.

[34] Singh I, Tripathi VS (2011). Micro strip patch antenna and its applications: a survey. Int J Comput Appl Technol.

[35] Kalaiselvi K, Suresh GR, Ravi V (2019). An efficient approach for the detection of link failures in WBAN system for health care applications. Int J Commun Syst.

[36] Khan JY, Yuce MR, Bulger G, Harding B (2012). Wireless Body Area Network (WBAN) design techniques and performance evaluation. J Med Syst.

[37] Sun M, Kiourti A, Wang H, Zhao S, Zhao G, Lu X (2016). Enhanced microwave hyperthermia of cancer cells with fullerene. Mol Pharm.

[38] Jhajharia T, Tiwari V, Bhatnagar D, Yadav D, Rawat S (2018). A dual-band CP dual-orthogonal arms monopole antenna with slanting edge DGS for C-band wireless applications. Int J Electron Commun.

[39] Nova IC, Derrington IM, Craig JM, Noakes MT, Tickman BI, Doering K (2017). Investigating asymmetric salt profiles for nanopore DNA sequencing with biological porin MspA. PLoS One.

[40] Kawate T, Gouaux E (2003). Arresting and releasing Staphylococcal alpha-hemolysin at intermediate stages of pore formation by engineered disulfide bonds. Protein Sci.

[41] Wang S, Zhao Z, Haque F, Guo P (2018). Engineering of protein nanopores for sequencing, chemical or protein sensing and disease diagnosis. Curr Opin Biotechnol.

[42] Guan X, Gu LQ, Cheley S, Braha O, Bayley H (2005). Stochastic sensing of TNT with a genetically engineered pore. Chembiochem.

[43] Aksakal S, Aksakal R, Becer CR (2018). Thioester functional polymers. Polym Chem.

[44] Liu L, Wu L, Tan J, Wang L, Liu Q, Liu P (2015). “Reduction” responsive thymine-conjugated biodynamers: synthesis and solution properties. Polym Chem.

[45] Wang S, Zhou Z, Zhao Z, Zhang H, Haque F, Guo P (2017). Channel of viral DNA packaging motor for real time kinetic analysis of peptide oxidation states. Biomaterials.

[46] Zhou Z, Ji Z, Wang S, Haque F, Guo P (2016). Oriented single directional insertion of nanochannel of bacteriophage SPP1 DNA packaging motor into lipid bilayer via polar hydrophobicity. Biomaterials.

[47] Monge F, Fanni A, Jiang S, Whitten DG, Bhaskar K, Chi EY (2019). Luminescent molecular sensors for the selective detection of neurodegenerative disease protein pathology in CSF. Biophys J.

[48] Movileanu L, Howorka S, Braha O, Bayley H (2000). Detecting protein analytes that modulate transmembrane movement of a polymer chain within a single protein pore. Nat Biotechnol.

[49] Wang S, Zhao Z, Haque F, Guo P (2018). Engineering of protein nanopores for sequencing, chemical or protein sensing and disease diagnosis. Curr Opin Biotechnol.

[50] Ho CW, Van Meervelt V, Tsai KC, De Temmerman PJ, Mast J, Maglia G (2015). Engineering a nanopore with co-chaperonin function. Sci Adv.

[51] Wang J, Boisvert DC (2003). Structural basis for GroEL-assisted protein folding from the crystal structure of (GroEL-KMgATP)14 at 20A resolution. J Mol Biol.

[52] Trzpis M, McLaughlin PM, de Leij LM, Harmsen MC (2007). Epithelial cell adhesion molecule: more than a carcinoma marker and adhesion molecule. Am J Pathol.

[53] Fernandez M, Espinosa HG, Thiel DV, Arrinda A (2018). Wearable slot antenna at 245 GHz for off-body radiation: analysis of efficiency, frequency shift, and body absorption. Bioelectromagnetics.

[54] Winnacker M (2017). Polyamides and their functionalization: recent concepts for their applications as biomaterials. Biomater Sci.

[55] Karthik V, Rama Rao T (2017). Investigations on SAR and thermal effects of a body wearable microstrip antenna. Wirel Pers Commun.

[56] Babaeva NY, Kushner MJ (2010). Intracellular electric fields produced by dielectric barrier discharge treatment of skin. J Phys D Appl Phys.

[57] Wahlsten O, Apell P (2019). Wounds as probes of electrical properties of skin. J Electr Bioimpedance.

[58] Shrestha S, Lee SR, Choi D-Y (2014). A new fractal-based miniaturized dual band patch antenna for RF energy harvesting. Int J Antennas Propag.

[59] Park SI, Brenner DS, Shin G, Morgan CD, Copits BA, Chung HU (2015). Soft, stretchable, fully implantable miniaturized optoelectronic systems for wireless optogenetics. Nat Biotechnol.

[60] Ibraheem AA, Manteghi M (2014). Performance of an implanted electrically coupled loop antenna inside human body. Prog Electromagn Res.

[61] Farrell BJ, Prilutsky BI, Ritter JM, Kelley S, Popat K, Pitkin M (2014). Effects of pore size, implantation time, and nano-surface properties on rat skin ingrowth into percutaneous porous titanium implants. J Biomed Mater Res A.

[62] Luo L, Hu B, Wu J, Yan T, Xu LJ (2019). Compact dual-band antenna with slotted ground for implantable applications. Microw Opt Technol Lett.

[63] Kim J, Rahmat-Samii Y (2004). Implanted antennas inside a human body: simulations, designs, and characterizations. IEEE Trans Microw Theory Tech.

[64] Guo T, Leng W, Wang A, Li J, Zhang Q (2014). A novel planar parasitic array antenna with frequency-and pattern-reconfigurable characteristics. IEEE Antennas Wirel Propag Lett.

[65] Lee CM, Yo TC, Luo CH, Tu CH, Juang YZ (2007). Compact broadband stacked implantable antenna for biotelemetry with medical devices. Electron Lett.

[66] Duan Z, Guo YX, Xue RF, Je M, Kwong DL (2012). Differentially fed dual-band implantable antenna for biomedical applications. IEEE Trans Antennas Propag.

[67] Liu WC, Chen SH, Wu CM (2009). Bandwidth enhancement and size reduction of an implantable PIFA antenna for biotelemetry devices. Microw Opt Technol Lett.

[68] Chien TF, Cheng CM, Yang HC, Jiang JW, Luo CH (2010). Development of nonsuperstrate implantable low-profile CPW-fed ceramic antennas. IEEE Antennas Wirel Propag Lett.

[69] Xu LJ, Guo YX, Wu W (2013). Miniaturised slot antenna for biomedical applications. Electron Lett.

[70] Huo K, Xu N, Fu J, Chu PK. Bioactive inorganic-ion-doped titania nanotube coatings on bone implants with enhanced osteogenic activity and antibacterial properties. In: Subramani K, Ahmed W, eds. Nanobiomaterials in Clinical Dentistry. 2nd ed. Elsevier; 2019. p. 401-27. 10.1016/b978-0-12-815886-9.00017-6

[71] Zradziński P, Karpowicz J, Gryz K. Modelling the influence of the electromagnetic field on a user of a bone conduction hearing medical implant. In: Korbicz J, Maniewski R, Patan K, Kowal M, eds. Current Trends in Biomedical Engineering and Bioimages Analysis. Cham: Springer; 2019. p. 245-55. 10.1007/978-3-030-29885-2_22

[72] Gupta A, Kansal A, Chawla P (2019). Design of a patch antenna with square ring-shaped-coupled ground for on-/off body communication. Int J Electron.

[73] Li R, Li B, Du G, Sun X, Sun H (2019). A compact broadband antenna with dual-resonance for implantable devices. Micromachines (Basel).

[74] Jeong MJ, Hussain N, Park JW, Park SG, Rhee SY, Kim N (2019). Millimeter-wave microstrip patch antenna using vertically coupled split ring metaplate for gain enhancement. Microw Opt Technol Lett.

[75] Dogan H, Basyigit IB, Yavuz M, Helhel S (2019). Signal level performance variation of radio frequency identification tags used in cow body. International Journal of RF and Microwave Computer-Aided Engineering.

[76] Das D, Maity S, Chatterjee B, Sen S (2019). Enabling covert body area network using electro-quasistatic human body communication. Sci Rep.

[77] Ullah Z, Ahmed I, Razzaq K, Naseer MK, Ahmed N (2019). DSCB: Dual sink approach using clustering in body area network. Peer Peer Netw Appl.

[78] Mukherjee S, Amin R, Biswas GP (2019). Design of routing protocol for multi-sink based wireless sensor networks. Wireless Networks.

[79] Zradziński P, Karpowicz J, Gryz K (2019). Electromagnetic Energy Absorption in a Head Approaching a Radiofrequency Identification (RFID) Reader Operating at 1356 MHz in Users of Hearing Implants Versus Non-Users. Sensors (Basel).

[80] Rashid TB, Song HH (2019). Analysis of biological effects of cell phone radiation on human body using specific absorption rate and thermoregulatory response. Microw Opt Technol Lett.

[81] Wolynski JG, Sutherland CJ, Demir HV, Unal E, Alipour A, Puttlitz CM (2019). Utilizing Multiple BioMEMS sensors to monitor orthopaedic strain and predict bone fracture healing. J Orthop Res.

[82] Sheima Y, Caspari P, Opris DM (2019). Artificial muscles: dielectric elastomers responsive to low voltages. Macromol Rapid Commun.

[83] Luan Z, Liu L, Zong WH, Jin Z, Li S (2019). Design of an implantable antenna operating at ISM band using magneto-dielectric material. Prog Electromagn Res.

[84] Pakhomov YA, Rinkevich AB, Perov DV, Belyanin AF, Kuznetsov EA (2019). Dielectric permittivity of artificial crystals based on opal matrices with ZnO particles in the millimeter waveband. J Infrared Millim Terahertz Waves.

[85] Paul BK, Roy D, Manna S, Nandy P, Das S (2018). High dielectric response of cobalt aluminate mullite (CAM) nanocomposite over cobalt aluminate mullite polymer (CAMP) nanocomposite in PVDF matrix. J Electroceram.

[86] Maity S, Barman KR, Bhattacharjee S (2018). Silicon-based technology: circularly polarized microstrip patch antenna at ISM band with miniature structure using fractal geometry for biomedical application. Microw Opt Technol Lett.

[87] Scarpello ML, Kurup D, Rogier H, Ginste DV, Axisa F, Vanfleteren J, Joseph W, Martens L, Vermeeren G (2011). Design of an implantable slot dipole conformal flexible antenna for biomedical applications. IEEE T Antenn Propag.

[88] Neebha TM, Nesasudha M, Chrysolite E. Computational modeling and parametric analysis of an implantable patch antenna using finite-difference time-domain algorithm. In: Satapathy S, Bhateja V, Das S, eds. Smart Intelligent Computing and Applications. Singapore: Springer; 2019. p. 107-20. 10.1007/978-981-13-1921-1_11

[89] Patil KS, Rufus E (2019). A review on antennas for biomedical implants used for IoT based health care. Sens Rev.

[90] Lorenzatti Hiles G, Cates AL, El-Sawy L, Day KC, Broses LJ, Han AL (2019). A surgical orthotopic approach for studying the invasive progression of human bladder cancer. Nat Protoc.

[91] Das S, Islam H, Bose T, Gupta N (2019). Ultra wide band cpw-fed circularly polarized microstrip antenna for wearable applications. Wirel Pers Commun.

[92] Das TT, Vinayak S, Pai SN. Design of an energy harvesting system for wireless power transmission using microstrip antenna. In: Ray K, Sharan S, Rawat S, Jain S, Srivastava S, Bandyopadhyay A, eds. Engineering Vibration, Communication and Information Processing. Singapore: Springer; 2019. p. 481-94. 10.1007/978-981-13-1642-5_43

[93] Singh R, Seth D, Rawat S, Ray K. Performance investigations of multi-resonance microstrip patch antenna for wearable applications. In: Ray K, Sharma T, Rawat S, Saini R, Bandyopadhyay A, eds. Soft Computing: Theories and Applications. Singapore: Springer; 2019. p. 159-69. 10.1007/978-981-13-0589-4_15

[94] Soontornpipit P, Furse CM, Chung YC (2005). Miniaturized biocompatible microstrip antenna using genetic algorithm. IEEE Trans Antennas Propag.

[95] Xia W, Saito K, Takahashi M, Ito K (2009). Performances of an implanted cavity slot antenna embedded in the human arm. IEEE Trans Antennas Propag.

[96] Karacolak T, Hood AZ, Topsakal E (2008). Design of a dual-band implantable antenna and development of skin mimicking gels for continuous glucose monitoring. ‎IEEE Trans Microw Theory Tech.

[97] Alires SL, Monge FA, Whitten DG, Chi EY (2019). Novel Sensors for detecting Alzheimer’s disease related tau protein aggregates. Biophys J.

[98] Li X. Body Matched Antennas for Microwave Medical Applications. Vol 72. KIT Scientific Publishing; 2014.

[99] Srinivasan D, Gopalakrishnan M (2019). Breast cancer detection using adaptable textile antenna design. J Med Syst.

[100] Suppli NP, Deltour I, Damkjær LH, Christensen J, Jensen AB, Kroman NT (2011). Factors associated with the prescription of antidepressive medication to breast cancer patients. Acta Oncol.

[101] Çalışkan R, Gültekin SS, Uzer D, Dündar Ö (2015). A microstrip patch antenna design for breast cancer detection. Procedia Soc Behav Sci.

[102] Singh I, Tripathi VS, Tiwari S (2012). Dual band suspended microstrip right-angled isosceles triangular patch antenna. J Commun Eng Syst.

[103] Kaur A, Kaur A. Monostatic radar based ultra-wideband microwave imaging system featuring a miniature fork shaped microstrip patch antenna with a reduced DGS for early breast tumor detection. In: Choudhury S, Mishra R, Mishra R, Kumar A, eds. Intelligent Communication, Control and Devices. Singapore: Springer; 2020. p. 113-122. 10.1007/978-981-13-8618-3_13

[104] Inum R, Rana MM, Shushama KN, Quader MA (2018). EBG based microstrip patch antenna for brain tumor detection via scattering parameters in microwave imaging system. Int J Biomed Imaging.

[105] Wang L (2018). Microwave sensors for breast cancer detection. Sensors (Basel).

[106] Manoufali M, Bialkowski K, Mohammed B, Mills PC, Abbosh A (2019). Compact implantable antennas for cerebrospinal fluid monitoring. IEEE Trans Antennas Propag.

[107] Li H, Guo YX, Xiao SQ (2016). Broadband circularly polarised implantable antenna for biomedical applications. Electron Lett.

[108] Zhang X, Yan J, Zhang H, Chen Y (2019). Miniaturized substrate integrated waveguide 5G LTCC bandpass filter exploiting capacitive loaded cavities. International Journal of RF and Microwave Computer-Aided Engineering.

[109] Zhao X, Zhang J, Fan K, Duan G, Schalch J, Keiser GR (2019). Real-time tunable phase response and group delay in broadside coupled split-ring resonators. Phys Rev B.

[110] Van Trinh T, Jung CW (2019). Bandwidth and directivity enhancement of an internal folded monopole antenna loaded by interdigital capacitor for ultra-high-definition television applications. Microw Opt Technol Lett.

[111] Lazzi G, Lee R, Nikita KS (2019). Guest editorial for the special issue on wireless real-time health monitoring technology for personalized medicine. IEEE Trans Antennas Propag.

